# Arsenic in Phthisis

**Published:** 1869-04

**Authors:** M. Herard


ARSENIC IN PHTHISIS.—By M. HERARD, in *Medical Times and Gazette*, November 14.

At the last meeting of the Academie de Medecine, M. Herard read a report on a memoir presented by M. Moutard-Martin, "On the Value of Arsenic in the Treatment of Phthisis." The reporter observed that the question of the efficacy of arsenic in this disease had been frequently treated of, but that the observations hitherto published were defective in exactitude. Such a reproach cannot be directed against the present author, who, long accustomed to clinical practice, has taken care to furnish exact indications not only to the nature, but the stage of the disease, and the particular form it exhibited.

Moreover, he has detailed the effects of the arsenic treatment employed exclusively. Almost all the patients, after a few days' treatment, exhibited a marked improvement in their general condition. The appetite improves, the strength returns, the complexion is clearer, and the eye is more animated; and, at the end of three weeks or a month, flesh begins to be gained. The local malady undergoes less change, but even this is sometimes sensibly modified. The favorable action of the arsenic is most observed in cases in which there is no acute fever or serious digestive disturbance. When there is intense febrile heat, whether continuous or remittent, and this is accompanied by much sweating, vomiting, and diarrhœa, and especially when the phthisis is acute, there is little or no amelioration, or this is not durable. The results have been found to be much more favorable among patients in private practice, who are placed in better hygienic condition than in hospital patients. The first appreciable effect of the administration of arsenic is the return of the appetite; whether this results from its direct action on the mucous membrane of the stomach, or from its more general operation as a tonic and neurosthenic. Another important effect it produces is that it moderates the oxidization of tissue, and thus efficaciously opposes denutrition. M. Lolliot, the most recent observer, finds that the daily administration of ten milligrammes of arsenious acid produces a diminution of temperature, and a very notable diminution in the amount of urea. The reporter is inclined to believe that arsenic may exert a direct action on the lung, as the respiratory mucous membrane is one of the channels of its elimination, while it proves useful in chronic bronchitis and asthma, and is resorted to by the peasantry in the Tyrol and elsewhere to facilitate their respiration during the ascent of high mountains. The arsenious acid should be administered in the form of pills or granules, each containing one milligramme, seven or eight of which may be taken at first per diem, rapidly increasing the number to ten, or fifteen, and, very rarely, even to two centigrammes. By taking care so to divide the doses as not to give more than two pills at a time, and that when possible before meals, all accidents are avoided. It is only very rarely that it becomes necessary to suspend the medicine or diminish its dose on account of slight temporary gastro-intestinal disturbance. Still it is necessary, as a matter of precaution, from time to time to suspend the treatment. This important memoir, the reporter observes in conclusion, will greatly contribute to establish the entire harmlessness of this substance when properly administered, as well as its indubitable efficacy.

